# Development and Application of a TaqMan RT-qPCR for the Detection of Foot-and-Mouth Disease Virus in Pigs

**DOI:** 10.3390/vetsci11110541

**Published:** 2024-11-05

**Authors:** Changying Dong, Xingyu Xiao, Meiqi Wang, Yajuan Sun, Hui Jin, Yongzhe Zhang, Hongri Zhao, Qianyue Cao, Yanran Yang, Rui Yin

**Affiliations:** 1 College of Biological and Pharmaceutical Engineering, Jilin Agricultural Science and Technology University, Jilin 132101, China; dongchangying@jlnku.edu.cn (C.D.); xyxiao23@mails.jlu.edu.cn (X.X.); wangmeiqi@jlnku.edu.cn (M.W.); sunyaj@jlu.edu.cn (Y.S.); jinhui0726@jlu.edu.cn (H.J.); zhangyongzhe@jlnku.edu.cn (Y.Z.); zhaohongri@jlnku.edu.cn (H.Z.); caoqianyue@jlnku.edu.cn (Q.C.); yangyanran@jlnku.edu.cn (Y.Y.); 2 Department of Neurology, China-Japan Union Hospital of Jilin University, Changchun 130033, China; 3 College of Veterinary Medicine, Jilin Agricultural University, Changchun 130118, China

**Keywords:** foot-and-mouth disease virus, TaqMan real-time RT-qPCR, system optimization, detection methods

## Abstract

The global livestock industry is facing a serious threat from a widespread foot-and-mouth disease virus (FMDV) epidemic. The timely detection of FMDV can significantly mitigate its harmful effects. This study aimed to establish and evaluate a TaqMan fluorescence quantitative PCR assay to assess its sensitivity, specificity, reproducibility, and stability. The standard curve equation range is 6.43 × 10^9^–6.43 × 10^1^ copies/µL, with an R^2^ value of 0.996 and a standard curve equation of y = −3.586x + 36.245. The method successfully detected 64.3 copies/µL of the target gene for FMDV and exhibited high specificity for FMDV. Repeatability tests demonstrated low coefficients of variation within and between groups (<2%), indicating good reproducibility. The clinical samples analyzed using this method showed results consistent with those of the SYBR Green I RT-qPCR assay, confirming the reliability of the method. Overall, the developed test method displayed high sensitivity, specificity, reproducibility, and stability, making it suitable for the rapid diagnosis of foot-and-mouth disease in clinical settings.

## Simple Summary

Foot-and-mouth disease (FMD), caused by the foot-and-mouth disease virus (FMDV), is a prevalent infectious disease in swine. Timely detection and isolation of pigs infected with this virus are among the most effective strategies to prevent the disease's spread and mitigate potential economic damage. We have established a set of RT-qPCR detection methods for FMDV, which offer significant advantages. Notably, this detection method can effectively identify foot-and-mouth disease virus even in the presence of both exogenous and endogenous interfering substances. Furthermore, it exhibits high sensitivity, strong specificity, and good repeatability, enabling 100% detection of FMDV.

## 1. Introduction

Foot-and-mouth disease (FMD) is a highly infectious disease that affects variable domestic animals such as pigs, cattle, sheep, and other cloven-hoofed animals, both domesticated and wild [1,2]. Widespread FMD has led to significant economic losses in the livestock industry. Clinical symptoms of FMD include fever, loss of appetite, lameness, and blisters on the mouth, tongue, or teats, or between the hooves [3,4]. Although the mortality rate of FMD is low, it can cause persistent lameness and decreased reproductive ability in affected animals, even after recovery [5]. In the swine industry, distinguishing foot-and-mouth disease virus (FMDV) from other similar vesicular diseases such as Senecavirus A, swine vesicular disease virus (SVDV), and vesicular stomatitis virus can be challenging in early clinical practice [6]. FMDV is a 30-nm, non-enveloped icosahedral virus with a positive-sense, single-stranded RNA genome of approximately 8300 nucleotides [7,8]. The viral genome contains one open reading frame that encodes four structural proteins and at least eight non-structural proteins [9,10]. The development and application of the FMDV vaccine face many challenges because there is no cross-protection between the seven distinct serotypes (O, A, Asia1, C, SAT-1, SAT-2, and SAT-3) and many subtypes [10,11,12]. Therefore, laboratory tests are essential in cases of suspected FMDV infection.

Various detection methods for FMDV include virus isolation and identification, an enzyme-linked immunosorbent assay (ELISA), colloidal gold immunochromatography, RT-PCR, and RT-qPCR [13]. Virus isolation and identification are considered the “gold standard” for detecting viruses [14]. Processed disease materials are inoculated into cell cultures, followed by identification through neutralization tests. This method offers high specificity and can determine both the presence and serotype of the virus [15,16]. However, it is time-consuming, has stringent requirements regarding laboratory conditions and personnel, and exhibits low sensitivity [17,18]. ELISA identifies viruses through the interaction of antigens and antibodies. The procedure is relatively straightforward, making it suitable for testing a large number of samples and allowing for automation [19]. However, it is important to note that ELISA may produce false positive or false negative results, and it is less effective in detecting viral antigens during the early stages of infection [20,21]. Colloidal gold immunochromatography technology employs detection and quality control lines on the test strip to assess color development resulting from the interaction between an antigen and antibody with colloidal gold. This method is user-friendly, does not necessitate complex instrumentation, and provides rapid detection capabilities [22]. While it is well suited for prompt on-site testing, its sensitivity is relatively low. Consequently, the qualitative test results may be slightly less accurate and are primarily intended for preliminary screening [22,23]. After RT-PCR amplifies the sample, DNA gel electrophoresis is employed to ascertain the presence of viral RNA [24]. However, this method cannot be quantitatively detected [25]. Additionally, aerosol contamination can easily occur during this process, leading to false positive results [4,26].

RT-qPCR operates on principles similar to those of RT-PCR. In addition to the high specificity and sensitivity characteristic of RT-PCR, RT-qPCR also offers the capability to accurately quantify viral load [27,28]. Consequently, RT-qPCR technology plays a crucial role in the routine diagnosis of FMDV and has established itself as the current standard method for the rapid detection of this disease. Notably, during the RT-qPCR amplification process, the quality of the amplification template directly influences the reliability of the test results. In real sample testing, amplified samples frequently contain various interfering substances that can adversely affect the results of qPCR reactions. These substances may inhibit DNA polymerase, disrupt the binding of polymerase to primers and templates, and alter the concentration of Mg^2+^ [29,30,31,32]. However, this potential issue has not been noted in previous FMDV qPCR detection systems [32,33].

In this study, we developed a set of universal primers and probes based on the conserved gene sequences of seven different FMDV serotypes. Additionally, we optimized the annealing temperature and primer/probe concentrations to establish a highly specific FMDV RT-qPCR assay with a minimum detection limit of 64.3 copies/µL. Our method demonstrated reproducibility and excellent stability in the presence of various interfering substances, and it was able to specifically detect FMDV in clinical samples with low viral loads.

## 2. Materials and Methods

### 2.1. Samples and Materials

FMDV-inactivated vaccine, weakly virulent classical swine fever virus (CSFV) vaccine strain (HCLV), and weakly virulent porcine reproductive and respiratory syndrome virus (PRRSV) vaccines were purchased from different companies. Wild virulent strains of CSFV and porcine somatic cellular DNA/human whole genome were extracted, isolated, and preserved in a laboratory. The analytical-grade ethanol and isopropanol (purity ≥ 99.7%) used in this experiment were sourced from Samson Chemical Technology (Shanghai) Limited Company (Shanghai, China).

A total of 243 pig-derived clinical samples were collected from slaughterhouses, markets, and pig breeding sites. These samples included 65 tissue samples (lymph, lung, spleen, liver, kidney, and tonsils), 93 blood samples, 42 stool samples, 23 subsamples (throat swabs and anal swabs), and 20 environmental samples.

### 2.2. Nucleic Acid Extraction and Reverse Transcription

Total DNA and RNA were extracted from 200 µL/0.2 g of viral vaccine or clinical samples using the Viral RNA/DNA Isolation Kit (Sairuisi, Jinlin, China) following the provided instructions and then eluted with 50 µL of elution buffer. The extracted RNA was reverse-transcribed to synthesize cDNA using the M-MuLV First Strand cDNA Synthesis Kit (Shenggong, Shanghai, China) according to the manufacturer’s guidelines. The resulting cDNA and DNA were stored at −80 °C until needed.

### 2.3. Primers and Probes

Whole-genome sequences of each genotypic isolate of FMDV were downloaded from the National Center for Biotechnology Information. These sequences were analyzed using Vector NTI Advance 11.0 (Ink/Thermo Fisher Scientific, Carlsbad, CA, USA) for multiple sequence comparisons to identify conserved regions among the FMDV strains. Primers and probes were designed using DNA STAR software 11.0 (Madison, WI, USA), and specificity was verified using an online website (Supplementary Material S1). The primer and probe sequences are listed in Table 1. The primers and probes were synthesized via Bioengineering (Shanghai) Biological Engineering Company Limited and stored at −20 °C for future use.

### 2.4. Construction and Characterization of Standard Plasmids

We used the cDNA of the FMDV virus as a template and selected primers to amplify the target gene using conventional PCR. Following gel electrophoresis of 10 µL of the PCR product on a 1.5% gel, the fragment length was visually inspected on a gel imager to confirm its match with the target fragment length. Subsequently, the gel was cut to isolate the corresponding fragment size, which was purified using a gel recovery kit (Shenggong, Shanghai, China). The purified target fragment was ligated to the vector plasmid pUC57 using the pUC57-simpleEVL Seamless Cloning Kit (Biomed, Beijing, China), following the manufacturer’s instructions. The resulting positive clone was sent to Bioengineering Co., Ltd. (Shanghai, China), for sequencing and labeled as the FMDV plasmid. Sequencing was performed successfully. Standard plasmids positive for FMDV were extracted using a plasmid DNA extraction kit (Sairuisi, Jilin, China). The plasmid concentration was determined and converted to copy numbers using a specific formula. The recombinant plasmid was then serially diluted and stored at −20 °C for subsequent testing. The copy number was calculated as follows [18,19]:

copies/mL = 6.02 × 10^23^ (copies/mol) × (concentration g/mL)/(MW g/mol)

### 2.5. Preliminary Establishment of FDMV RT-qPCR

The reaction system for SYBR Green I qPCR included 12.5 µL of qPCR mixture, 0.5 µL each of forward and reverse primers, 1 µL of cDNA template, and 10.5 µL of double-distilled water in a total volume of 25 µL. The reaction conditions involved pre-denaturation at 95 °C for 5 min, followed by denaturation at 95 °C for 20 s, annealing at 60 °C for 30 s, extension at 68 °C for 30 s, 40 cycles, and final extension at 68 °C for 5 min.

For the TaqMan probe method, the qPCR reaction system included 1 µL of enzyme mix (Yisheng, Shanghai, China), 12.5 µL of PCR buffer, 0.5 µL each of forward and reverse primers, 0.1 µL of probe, and 5 µL of RNA template, with double-distilled water added up to 25 µL. The PCR reaction conditions were as follows: 50 °C, 10 s; 95 °C, 5 min; 95 °C, 15 s; 62 °C 30 s; and 45 cycles.

### 2.6. Optimization of RT-qPCR Reaction System

The FMDV virus plasmid was diluted 10 times to concentrations of 6.43 × 10^6^, 6.43 × 10^5^, and 6.43 × 10^4^ copies/µL. Three replicates were prepared for each concentration as a positive control, and sterile water was used as a negative control. Follow the recommended primer and probe concentrations of Hifair^®^ V C58P2 Multiplex One-Step RT-qPCR Probe Kit (UDG Plus), different dosages of FMDV F1/R1 (50, 70, 90, and 100 µM) and FMDV P2 (10, 20, 30 µM, and 50 µM) were tested for optimization. The instruction manual was followed to collect the fluorescence signals from the CY5 channel. The optimal primer and probe concentrations were determined by analyzing the PCR amplification curves and Ct values. Additionally, three different annealing temperatures (58 °C, 60 °C, and 62 °C) were tested in the RT-qPCR reaction to further optimize the reaction conditions.

### 2.7. Establishment of the Standard Curve of RT-qPCR

To establish the standard curve, the original concentration of the FMDV plasmid was diluted to 6.43 × 10^1^ copies/µL using a 10-fold gradient. The plasmid concentrations ranging from 6.43 × 10^1^ to 6.43 × 10^8^ copies/µL were used as templates for the real-time fluorescence PCR reaction system. PCR amplification was performed according to the established optimal reaction systems and conditions. The logarithm of the copy number was plotted on the x-axis, and the Ct value was plotted on the y-axis to create a standard curve for FMDV.

### 2.8. Limit of Detection Determination

To determine the lowest detection limit of the experimental research method, plasmid dilutions ranging from 6.43 × 10^2^ to 6.43 × 10^9^ copies/µL were used as positive templates, while sterile water served as the negative control. TaqMan fluorescence quantitative RT-qPCR was performed using an optimal reaction system. The lowest detection limit was determined, and the results were processed for calculation.

### 2.9. Specificity of FMDV RT-qPCR 

The FMDV recombinant plasmid was used as a positive control, and the DNA/RNA of common pig-infected CSFV, CSFV HCLV, FMDV, PRRSV, porcine cirovirus type 2, porcine cirovirus type 3, porcine epidemic diarrhea virus, and blood-borne and porcine parvovirus served as templates. Sterile deionized water was used as the negative control. TaqMan fluorescence quantitative RT-qPCR was conducted under optimized reaction conditions to assess the specificity of the established detection method.

### 2.10. Repeatability Test

To assess the reproducibility of the test, standard plasmids at concentrations of 6.43 × 10^3^, 6.43 × 10^5^, and 6.43 × 10^7^ copies/µL were selected. Three parallel experiments were performed for each concentration using an optimized qPCR reaction system and conditions to evaluate intragroup variation. Additionally, three fluorescence quantitative tests were performed at different time points for intergroup variability analysis. The mean and Ct values of each test batch were recorded, and the coefficients of variation were calculated.

### 2.11. Interference Assay

In this study, a recombinant plasmid at a concentration of 6.43 × 10^5^ copies/µL was combined with various interfering reagents (blood of sheep, liver tissues, intestinal contents, lung tissues, milk, mucin, oral swab, manure, animal feed, and reagents 1–4) in equal amounts (100 µL) to investigate their impact on detection results. The recombinant plasmid served as the positive control, whereas sterile water served as the negative control. RT-qPCR was conducted under optimal conditions. Each interference reagent was tested at least three times, with two samples taken to minimize sampling variability. Inhibition was defined as a 5% increase in the Ct value compared with the positive control, while significant inhibition was indicated by a 10% increase in the Ct value.

### 2.12. Detection of Clinical Samples

RNA was extracted from 100 clinical samples of porcine origin in accordance with the current standard GB/T 18935-2018 (https://std.cahec.cn/gb/details/412.html, accessed on 11 March 2024) for Foot and Mouth Disease Diagnostic Technology. The FMVD TaqMan RT-qPCR method developed in this study was employed for detection, and the outcomes were compared with those of SYBR Green I RT-qPCR to determine the respective positive and negative rates. A Ct value below 42 was considered positive, whereas a value above 42 was considered negative.

## 3. Results

### 3.1. Identification of Recombinant Plasmids

After the retrieval of the target strip of the FMDV virus, it was integrated into the PUC57-T vector to form a recombinant plasmid, referred to as the FMDV plasmid. Subsequent sequencing confirmed that the inserted fragment matched the reference sequence (TTCCTCAAAAGACACTTCCACATGGGACTACGGAACTGGGTTTTACAAACCTGTGATGGCCTCGAAGACCCTCGAGGCCATCCTCTCCTTTGCACGCCGTG). The FMDV plasmid was isolated, and its concentration was determined to be 198 ng/µL using a UV spectrophotometer, with a calculated copy number of 6.43 × 10^10^ copies/µL. The recombinant plasmid was successfully constructed.

### 3.2. Primer Probe Selection

**Figure 1:**
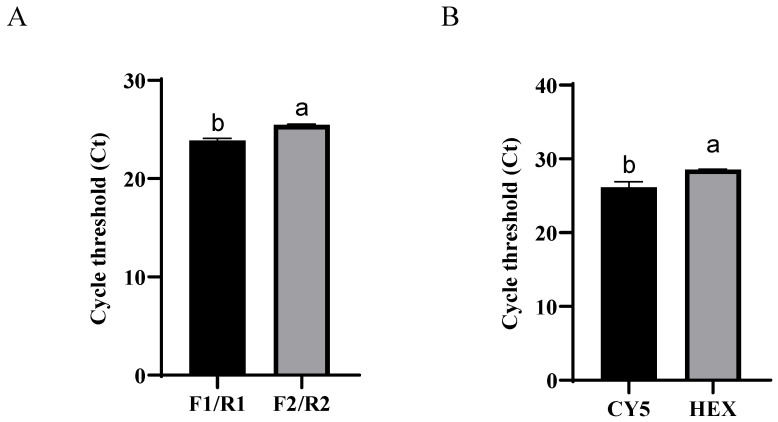
Selection of primers (**A**) and probes (**B**) for FMDV. Different letters indicate significant differences between groups (*p* < 0.05).

**Figure 2:**
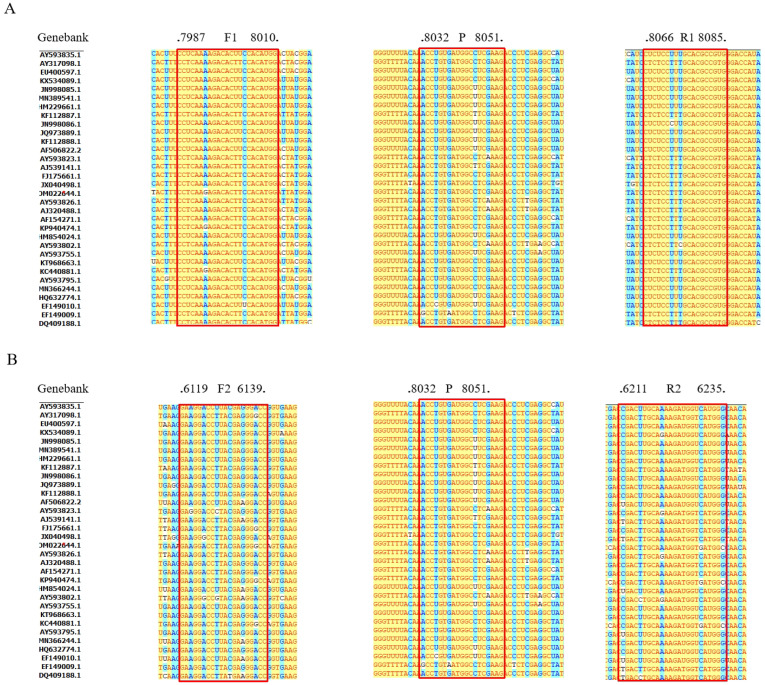
Alignments of coding sequences of reference viral strains deposited in GenBank. Sequences of F1/R1/P1 (**A**) and F2/R2/P2 (**B**). The red boxes indicate the positions of primers and probes.

FMDV plasmid DNA at a concentration of 6.43 × 10^5^ copies/µL was utilized as the amplification template. Two pairs of primers (Table 1) were evaluated using SYBR Green I based on Ct values (Figure 1A). The Ct values for the primers were as follows: F1/R1 (23.89 ± 0.21) < F2/R2 (25.46 ± 0.08). F1/R1 was selected as the primer, and the fluorescent groups of the probe were assessed using the TaqMan probe method. As depicted in Figure 1B, the probe with CY5 demonstrated superior amplification, with a Ct value of 26.17 ± 0.74. The positions of the primers and probes in the sequences are shown in Figure 2.

### 3.3. RT-qPCR Reaction System Optimization

Plasmids with concentrations of 6.43 × 10^6^, 6.43 × 10^5^, and 6.43 × 10^4^ copies/µL were used as templates for the experiment. Primer and probe concentrations, along with the annealing temperature, were optimized using F1/R1 as the primer and CY5 as the fluorescent probe label. According to Table 2, the most effective RT-qPCR amplification was achieved with 50 nM each of forward and reverse primers for FMDV, 10 nM of the probe, and an annealing temperature of 62 °C.

### 3.4. Establishment of the Standard Curve

**Figure 3:**
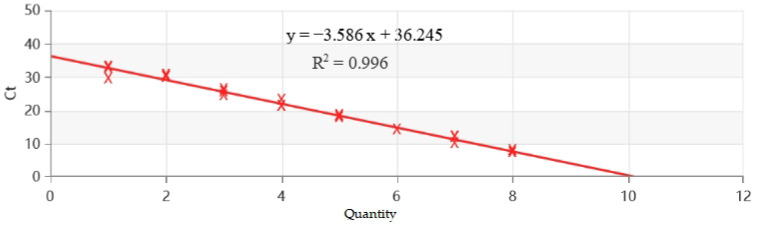
Standard curve for RT-qPCR of FMDV.

The prepared standard positive plasmids of FMDV were used as templates for a 10-fold multiplicative dilution, ranging in concentrations from 6.43 × 10^2^ to 6.43 × 10^9^ copies/µL. These templates were assayed using an established RT-qPCR system. The results revealed a standard curve equation of y = −3.586x + 36.245, with a coefficient of determination (R^2^) of 0.996, demonstrating a strong linear relationship between the concentrations of the standards (Figure 3).

### 3.5. Sensitivity Test of FMDV RT-qPCR

**Figure 4:**
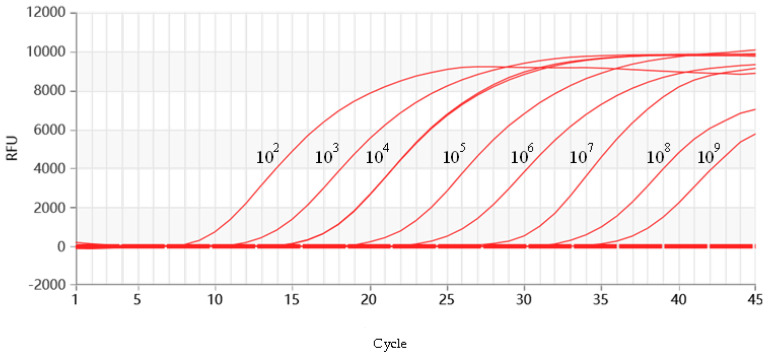
Sensitivity assay for RT-qPCR of FMDV. 10^2^–10^9^ represents the dilution ratio.

The standard recombinant positive plasmid of FMDV was diluted to various concentrations and tested using a multiplex RT-qPCR reaction system and reaction program. The results showed that FMDV could be detected at a minimum concentration of 64.3 copies/µL (Figure 4).

### 3.6. Specificity of the Detection System

**Figure 5:**
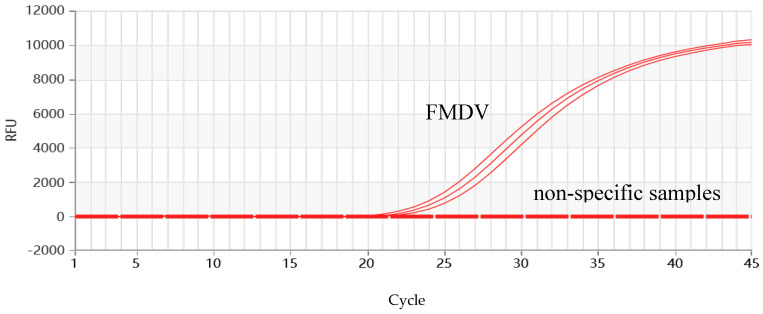
Specificity assay for RT-qPCR of FMDV.

The FMDV recombinant plasmid was used as the positive control, while the DNA/RNA of common pig-infected CSFV, CSFV HCLV, FMDV, PRRSV, PCV-2, PCV-3, PED, Bb, and PPV served as templates. Sterile deionized water was set as the negative control. TaqMan fluorescence quantitative RT-qPCR was conducted under optimized reaction conditions and systems to assess the specificity of the detection method established (Figure 5).

### 3.7. Repeatability Test of FMDV RT-qPCR

In this study, we examined the within- and between-group reproducibility of plasmid standards at three concentrations using an established method. The coefficients of variation for within-group and between-group were found to be 0.74–1.78% and 0.70–0.97%, respectively (Table 3). These values, both below 2%, suggested that the method demonstrated good reproducibility.

### 3.8. Interference Test of FMDV RT-qPCR

Recombinant plasmids containing various endogenous and exogenous interfering substances were used as templates for amplification and were analyzed using the developed FMDV RT-qPCR assay. Table 4 shows that, with the exception of reagent 3, manure, and milk inhibiting the FMDV plasmid and sheep blood causing significant inhibition, no other interfering reagents displayed inhibitory effects. These findings suggest that the FMDV RT-qPCR detection system established in this study is resilient to interfering substances and is suitable for detecting complex samples.

### 3.9. Clinical Sample Results

A total of 243 clinical samples were analyzed using the TaqMan RT-qPCR method established in this study. The results indicated that the positive rate of tissue samples was 7.93% (5/65), the positive rate of blood samples was 7.53% (7/93), the positive rate of stool samples was 7.14% (3/42), the positive detection rate of swab samples was 8.70% (2/23), and the positive rate of environmental samples was 5% (1/20). Additionally, 50 clinical samples were randomly selected and tested using SYBR Green qPCR, with results consistent with those obtained from TaqMan RT-qPCR, thereby demonstrating the high accuracy of this method.

## 4. Discussion

FMD is a highly contagious animal infection that has a significant impact on the global livestock industry, particularly on domestic swine breeding. Many countries prioritize FMD monitoring because of its potential to cause catastrophic outbreaks. Although virus isolation and culture are traditionally used as the gold standard for diagnosing FMDV [20,21,22], this method may lack sensitivity for samples with low viral loads and is dependent on the quality of sample collection. The lengthy incubation period required for viral isolation hinders its widespread use. Enzyme-linked immunosorbent assay, which detects FMDV through antigen-antibody binding, offers a faster and more versatile alternative to virus isolation [23,24]. However, the low sensitivity of enzyme-linked immunosorbent assays limits their large-scale application [25]. Currently, the PCR is the dominant approach to virus detection in molecular biology. The TaqMan RT-PCR assay has been endorsed by the World Organization for Animal Health (OIE) for the diagnosis of FMDV, owing to its rapid, accurate, and sensitive detection of nucleic acids resembling various pathogenic bacteria. This development is important for prompt clinical diagnosis, prevention, and epidemiological research, as other diagnostic technologies lack comparable capabilities.

The optimization of conventional qPCR systems typically focuses on adjusting the primer concentration, probe concentration, and annealing temperature [34,35,36]. In this study, we demonstrated that, by attaching different fluorescent reporter groups and fluorescent bursting groups to both ends of the probe, the use of varied fluorescent groups can yield distinct effects on detection results, even under identical primer and probe sequence conditions (Figure 1B). This finding opens new avenues for optimizing qPCR detection systems. Given that samples received in diagnostic laboratories for FMDV diagnosis are often complex and may contain various additives, including PCR inhibitors that can persist despite efforts to clean the samples, these factors significantly affect the efficacy of the qPCR examination system. This issue has not been noted in the development of previous FMDV qPCR detection systems [32,33,37,38]. In this study, we utilized an equal mixture of interfering reagents and FMDV as a template to analyze the differences in Ct values between the positive control and samples following the addition of interfering reagents. The results from these interference experiments indicated that the RT-qPCR assay system for FMDV established in this study exhibited greater tolerance to various types of interference, resulting in a reduced impact on FMDV diagnosis in the laboratory.

In this study, we developed specific primers and probes targeting the ORF5 of FMDV. The probe was labeled CY5 at the 5′ end for fluorescence and BHQ3 at the 3′ end for quenching. Via the optimization of the reaction conditions, the virus detection limit was achieved at 64.3 copies/µL, with a coefficient of variation of less than 2% for repeatability within and between groups. The universal FMDV RT-qPCR method presented here can be applied to a variety of clinical samples, including whole blood, tissue, lymph, throat swabs, and stool. It offers a specific, sensitive, and accurate approach to the clinical identification and epidemiological investigation of various FMDV genotypes.

Despite the advancements made in this study, several limitations remain. During the screening process for the fluorescence reporter group and the fluorescence quenching group of the probes, it was demonstrated that probes with different fluorescent groups influenced detection. However, only two fluorescent groups were compared, and the underlying reasons for this phenomenon were not further explored. Additionally, while the newly developed FMDV RT-qPCR exhibits improved tolerance to interfering substances, it still necessitates traditional multi-step nucleic acid extraction prior to qPCR amplification. This requirement constrains its effectiveness in field-based pathogen detection. Future research will aim to investigate molecular point-of-care testing (POCT) detection systems as a viable solution for field-based FMDV detection.

## Figures and Tables

**Table 1:** Nucleotide sequence of primers and probes for FMDV.

Primer/Probe	Sequences (5′-3′)	Length (bp)	Product Length (bp)
FMVD-F1	CCTCAAAAGACACTTCCACATGG	23	99
FMVD-R1	CTCTCCTTTGCACGCCGTG	19	
FMVD-F2	GAAGGACCTTACGAGGGACC	20	124
FMVD-R2	CGACTTGCAAAAGATGGTCATGGG	24	
FMVD-P1	HEX-CGAGGGTCTTCGAGGCCATCACAGGT-TAMRA	26	99
FMVD-P2	CY5-CGAGGGTCTTCGAGGCCATCACAGGT-BHQ3		

**Table 2:** Optimization of primer and probe concentration and annealing temperature.

Factor	Parameters	Plasmid Concentration (Copies/µL)		
		6.43 × 10^4^	6.43 × 10^5^	6.43 × 10^6^
Primer concentration (µM)	50	33.69 ± 0.55	29.76 ± 0.45	25.43 ± 0.32
	70	33.34 ± 0.41	30.11 ± 0.05	25.15 ± 0.24
	90	34.74 ± 0.69	31.24 ± 0.16	25.82 ± 0.25
	100	33.61 ± 1.48	30.09 ± 0.20	25.59 ± 0.44
Probe concentration (µM)	10	33.61 ± 0.62	28.63 ± 0.33	24.43 ± 0.23
	20	34.20 ± 0.94	29.78 ± 0.29	25.81 ± 0.78
	30	34.20 ± 0.94	29.78 ± 0.29	25.81 ± 0.78
	50	33.54 ± 0.34	29.77 ± 0.66	24.69 ± 0.13
Annealing temperature (℃)	58	24.98 ± 0.03	31.58 ± 0.99	35.12 ± 0.89
	60	25.15 ± 0.07	30.88 ± 0.73	34.21 ± 0.35
0.35	62	24.80 ± 0.01	30.36 ± 0.66	34.09 ± 0.39

**Table 3:** Reproducible of the real-time RT-qPCR assay (n = 3).

Concentrations of Standard Plasmid	Within-Group Variance		Between-Group Variance	
	Mean ± SD	CV (%)	Mean ± SD	CV (%)
6.43 × 10^7^ copies/µL	14.74 ± 0.26	1.78	14.85 ± 0.14	0.97
6.43 × 10^5^ copies/µL	24.82 ± 0.27	1.07	24.99 ± 0.19	0.75
6.43 × 10^3^ copies/µL	32.65 ± 0.24	0.74	32.32 ± 0.23	0.70

**Table 4:** Interference assay of the real-time qPCR assay.

Interference Reagents	Name	Ct (Mean ± SD)	CV (%)
Exogenous and endogenous interference with material	Blood of sheep	25.42 ± 1.44	5.67
	Liver tissues	23.44 ± 0.79	4.45
	Intestinal contents	21.42 ± 0.72	0.55
	Lung tissues	22.18 ± 0.62	1.71
	Milk	23.84 ± 0.94	3.35
	Mucin	21.94 ± 0.45	3.38
	Oral swab	22.48 ± 1.00	2.79
	Manure	24.31 ± 0.42	3.94
	Animal feed	21.01 ± 0.12	2.06
	Reagent 1 ^a^	21.93 ± 0.73	3.40
	Reagent 2 ^b^	22.49 ± 0.65	2.90
	Reagent 3 ^c^	23.81 ± 0.67	2.81
	Reagent 4 ^d^	22.81 ± 1.22	5.35
Positive control	Standard plasmid	22.59 ± 1.39	6.13
Negative control	ddH_2_O	N/A ^e^	N/A

^a^: equivalent mixture of cephalosporin, florfenicol, and tilmicosin; ^b^: equivalent mixture of amoxicillin, doxycycline, and tamoxifen; ^c^: equivalent mixture of dexamethasone and gentamicin; ^d^: equivalent mixture of ribavirin and amantadine; ^e^: N/A indicates not applicable.

## Data Availability

The data that support the findings of this study are available from the corresponding author upon reasonable request.

## Supplementary Materials

The following supporting information can be downloaded at: https://www.mdpi.com/article/10.3390/vetsci11110541/s1, Supplementary Material S1: F1 and R1 Alignment.

## References

[1] Hwang Y.J., Lee K.K., Kim J.W., Chung K.H., Kim S.J., Yun W.S., Lee C.S. (2021). Effective Diagnosis of Foot-And-Mouth Disease Virus (FMDV) Serotypes O and A Based on Optical and Electrochemical Dual-Modal Detection. Biomolecules.

[2] Shaw A.E., Burman A., Asfor A., Brocchi E., Grazioli S., Browning C., Ludi A., Tuthill T.J., King D.P. (2022). Avidity of Polyclonal Antibodies to Foot-and-Mouth Disease Virus in Bovine Serum Measured Using Bio-Layer Interferometry. Viruses.

[3] Pega J., Di Giacomo S., Bucafusco D., Schammas J.M., Malacari D., Barrionuevo F., Capozzo A.V., Rodríguez L.L., Borca M.V., Pérez-Filgueira M. (2015). Systemic Foot-and-Mouth Disease Vaccination in Cattle Promotes Specific Antibody-Secreting Cells at the Respiratory Tract and Triggers Local Anamnestic Responses upon Aerosol Infection. J. Virol..

[4] Banerjee T., Tummala T., Elliott R., Jain V., Brantley W., Hadorn L., Santra S. (2019). Multimodal Magneto-Fluorescent Nanosensor for Rapid and Specific Detection of Blood-Borne Pathogens. ACS Appl. Nano Mater..

[5] Arzt J., Belsham G.J., Lohse L., Bøtner A., Stenfeldt C. (2018). Transmission of Foot-and-Mouth Disease from Persistently Infected Carrier Cattle to Naive Cattle via Transfer of Oropharyngeal Fluid. mSphere.

[6] Zhang X., Zhu Z., Yang F., Cao W., Tian H., Zhang K., Zheng H., Liu X. (2018). Review of Seneca Valley Virus: A Call for Increased Surveillance and Research. Front. Microbiol..

[7] Robinson L., Windsor M., McLaughlin K., Hope J., Jackson T., Charleston B. (2011). Foot-and-mouth disease virus exhibits an altered tropism in the presence of specific immunoglobulins, enabling productive infection and killing of dendritic cells. J. Virol..

[8] Xu J., Qian P., Wu Q., Liu S., Fan W., Zhang K., Wang R., Zhang H., Chen H., Li X. (2014). Swine interferon-induced transmembrane protein, sIFITM3, inhibits foot-and-mouth disease virus infection in vitro and in vivo. Antivir. Res..

[9] Banda F., Sinkala Y., Mataa L., Lebea P., Sikombe T., Kangwa H.L., Fana E.M., Mokopasetso M., Wadsworth J., Knowles N.J. (2021). Characterization of Foot-and-Mouth Disease Viruses in Zambia-Implications for the Epidemiology of the Disease in Southern Africa. Viruses.

[10] Bari F.D., Parida S., Asfor A.S., Haydon D.T., Reeve R., Paton D.J., Mahapatra M. (2015). Prediction and characterization of novel epitopes of serotype A foot-and-mouth disease viruses circulating in East Africa using site-directed mutagenesis. J. Gen. Virol..

[11] Kotecha A., Zhang F., Juleff N., Jackson T., Perez E., Stuart D., Fry E., Charleston B., Seago J. (2016). Application of the thermofluor PaSTRy technique for improving foot-and-mouth disease virus vaccine formulation. J. Gen. Virol..

[12] Bronsvoort B.M., Parida S., Handel I., McFarland S., Fleming L., Hamblin P., Kock R. (2008). Serological survey for foot-and-mouth disease virus in wildlife in eastern Africa and estimation of test parameters of a nonstructural protein enzyme-linked immunosorbent assay for buffalo. Clin. Vaccine Immunol. CVI.

[13] Yang M., Zhmendak D., Mioulet V., King D.P., Burman A., Nfon C.K. (2022). Combining a Universal Capture Ligand and Pan-Serotype Monoclonal Antibody to Develop a Pan-Serotype Lateral Flow Strip Test for Foot-and-Mouth Disease Virus Detection. Viruses.

[14] Yu M., Huang P., Li Y., Song Y., Liu X., Feng N., Jin H., Bai Y., Zhang H., Li Y. (2022). A Visual Assay of a Loop-Mediated Isothermal Amplification Based Vertical Immunoassay for SARS-CoV-2 RNA Detection. Front. Microbiol..

[15] Muth D., Corman V.M., Meyer B., Assiri A., Al-Masri M., Farah M., Steinhagen K., Lattwein E., Al-Tawfiq J.A., Albarrak A. (2015). Infectious Middle East Respiratory Syndrome Coronavirus Excretion and Serotype Variability Based on Live Virus Isolates from Patients in Saudi Arabia. J. Clin. Microbiol..

[16] Tiwari S., Dhole T.N. (2018). Assessment of enteroviruses from sewage water and clinical samples during eradication phase of polio in North India. Virol. J..

[17] Ren N., Jinli J., Chen Y., Zhou X., Wang J., Ge P., Khan F.A., Zhang L., Hu C., Robertson I.D. (2018). Identification of new diagnostic biomarkers for Mycobacterium tuberculosis and the potential application in the serodiagnosis of human tuberculosis. Microb. Biotechnol..

[18] Wu K., Zhang Y., Zeng S., Liu X., Li Y., Li X., Chen W., Li Z., Qin Y., Chen J. (2021). Development and Application of RAA Nucleic Acid Test Strip Assay and Double RAA Gel Electrophoresis Detection Methods for ASFV and CSFV. Front. Mol. Biosci..

[19] Trombetta C.M., Remarque E.J., Mortier D., Montomoli E. (2018). Comparison of hemagglutination inhibition, single radial hemolysis, virus neutralization assays, and ELISA to detect antibody levels against seasonal influenza viruses. Influenza Other Respir. Viruses.

[20] Bivona A.E., Czentner L., Sanchez Alberti A., Cerny N., Cardoso Landaburu A.C., Nevot C., Estévez O., Marco J.D., Basombrio M.A., Malchiodi E.L. (2019). Recombinant Cysteine Proteinase B from Leishmania braziliensis and Its Domains: Promising Antigens for Serodiagnosis of Cutaneous and Visceral Leishmaniasis in Dogs. J. Clin. Microbiol..

[21] Liu L., Liu W., Zheng Y., Jiang X., Kou G., Ding J., Wang Q., Huang Q., Ding Y., Ni W. (2020). A preliminary study on serological assay for severe acute respiratory syndrome coronavirus 2 (SARS-CoV-2) in 238 admitted hospital patients. Microbes Infect..

[22] Shen H., Wan Y., Wu X., Zhang Y., Li J., Cui T., Sun H., Cui H., He K., Hui G. (2022). Hapten designs based on aldicarb for the development of a colloidal gold immunochromatographic quantitative test strip. Front. Nutr..

[23] Zhang P., Gao Q., Wang T., Ke Y., Mo F., Jia R., Liu W., Liu L., Zheng S., Liu Y. (2021). Development and evaluation of a serological test for diagnosis of COVID-19 with selected recombinant spike proteins. Eur. J. Clin. Microbiol. Infect. Dis. Off. Publ. Eur. Soc. Clin. Microbiol..

[24] Baek Y.H., Um J., Antigua K.J.C., Park J.H., Kim Y., Oh S., Kim Y.I., Choi W.S., Kim S.G., Jeong J.H. (2020). Development of a reverse transcription-loop-mediated isothermal amplification as a rapid early-detection method for novel SARS-CoV-2. Emerg. Microbes Infect..

[25] Englezou P.C., Sapet C., Démoulins T., Milona P., Ebensen T., Schulze K., Guzman C.A., Poulhes F., Zelphati O., Ruggli N. (2018). Self-Amplifying Replicon RNA Delivery to Dendritic Cells by Cationic Lipids. Mol. Ther. Nucleic Acids.

[26] Sun Y., Yu L., Liu C., Ye S., Chen W., Li D., Huang W. (2021). One-tube SARS-CoV-2 detection platform based on RT-RPA and CRISPR/Cas12a. J. Transl. Med..

[27] Baloch A.S., Liu C., Liang X., Liu Y., Chen J., Cao R., Zhou B. (2019). Avian Flavivirus Enters BHK-21 Cells by a Low pH-Dependent Endosomal Pathway. Viruses.

[28] Hashem M.A., Maetani F., Kayesh M.E.H., Eiei T., Mochizuki K., Ito A., Sakurai H., Asai T., Tsukiyama-Kohara K. (2020). Transmission of Koala Retrovirus from Parent Koalas to a Joey in a Japanese Zoo. J. Virol..

[29] Ruan H.T., Wang R.L., Li H.T., Liu L., Kuang T.X., Li M., Zou K.S. (2022). Effects of sampling strategies and DNA extraction methods on eDNA metabarcoding: A case study of estuarine fish diversity monitoring. Zool. Res..

[30] Guo L., Yang S.L., Wang C.D., Hou R., Chen S.J., Yang X.N., Liu J., Pan H.B., Hao Z.X., Zhang M.L. (2013). Phylogenetic analysis of the haemagglutinin gene of canine distemper virus strains detected from giant panda and raccoon dogs in China. Virol. J..

[31] Yi J., Wang N., Wu J., Tang Y., Zhang J., Zhu L., Rui X., Guo Y., Xu Y. (2021). Development of a Droplet Digital Polymerase Chain Reaction for Sensitive Detection of Pneumocystis jirovecii in Respiratory Tract Specimens. Front. Med..

[32] Wang Y., Das A., Zheng W., Porter E., Xu L., Noll L., Liu X., Dodd K., Jia W., Bai J. (2020). Development and evaluation of multiplex real-time RT-PCR assays for the detection and differentiation of foot-and-mouth disease virus and Seneca Valley virus 1. Transbound. Emerg. Dis..

[33] Biswal J.K., Ranjan R., Mohapatra J.K., Rout M., Joshi H.R., Singh R.P. (2023). Development of TaqMan Probe-Based One-Step RT-qPCR Assay Targeting 2B-NSP Coding Region for Diagnosis of Foot-and-Mouth Disease in India. Curr. Microbiol..

[34] Liu Q.l., Wang Y., Fang J.C.q.P.F.Y.Y.Z.h. (2024). Establishment of a TaqMan probe-based qPCR assay for detecting microsporidia Enterospora epinepheli in grouper. J. Fish Dis..

[35] Zhu H., Wang G., Liu X., Wu W., Yu T., Zhang W., Liu X., Cheng G., Wei L., Ni L. (2024). Establishment and application of a quadruplex real-time RT-qPCR assay for differentiation of TGEV, PEDV, PDCoV, and PoRVA. Microb. Pathog..

[36] Alonso-Rebollo A., Ramos-Gómez S., Busto M.D., Ortega N. (2017). Development and optimization of an efficient qPCR system for olive authentication in edible oils. Food Chem..

[37] Pinheiro-de-Oliveira T.F., Fonseca A.A., Camargos M.F., Laguardia-Nascimento M., de Oliveira A.M., Cottorello A.C.P., Goes-Neto A., Barbosa-Stancioli E.F. (2018). Development of a droplet digital RT-PCR for the quantification of foot-and-mouth virus RNA. J. Virol. Methods.

[38] El Bagoury G.F., Elhabashy R., Mahmoud A.H., Hagag N.M., El Zowalaty M.E. (2022). Development and evaluation of one-step real-time RT-PCR assay for improved detection of foot-and-mouth disease virus serotypes circulating in Egypt. J. Virol. Methods.

